# Active Adjustment of Surface Accuracy for a Large Cable-Net Structure by Shape Memory Alloy

**DOI:** 10.3390/ma12162619

**Published:** 2019-08-16

**Authors:** Xiangjun Jiang, Fengqun Pan, Yesen Fan, Jingli Du, Mingbo Zhu, Zhen Chen

**Affiliations:** 1 Key Laboratory of Electronic Equipment Structural Design, Xidian University, Xi’an 710071, China; 2 Xi’an Institute of Space Radio Technology, Xi’an 710100, China; 3 State Key Laboratory for Manufacturing System, Xi’an Jiaotong University, Xi’an 710049, China

**Keywords:** shape memory alloy, cable-net antenna structure, finite element modeling, active adjustment of surface accuracy

## Abstract

The high surface accuracy design of a cable-net antenna structure under the disturbance of the extremely harsh space environment requires the antenna to have good in-orbit adjustment ability for surface accuracy. A shape memory cable-net (SMC) structure is proposed in this paper and believed to be able to improve the in-orbit surface accuracy of the cable-net antenna. Firstly, the incremental stiffness equation of a one-dimensional bar element of the shape memory alloy (SMA) to express the relationship between the force, temperature and deformation was effectively constructed. Secondly, the finite element model of the SMC antenna structure incorporated the incremental stiffness equation of a SMA was established. Thirdly, a shape active adjustment procedure of surface accuracy based on the optimization method was presented. Finally, a numerical example of the shape memory cable net structure applied to the parabolic reflectors of space antennas was analyzed.

## 1. Introduction

Large deployable antennas (LDAs) are considered increasingly for present and future satellites in the fields of communication, electronic reconnaissance, navigation, remote sensing and telecommunication missions [1,2,3,4,5]. Cable-net LDAs, which is the most-anticipated type of LDAs, are expected to be used for future missions such as the soil moisture active passive [2] and reflector synergies between Telecom and Earth observation [4], which can obtain a proper balance between the surface accuracy and the acceptance ratio. The representative type of the cable-net antenna, the AstroMesh-like antenna, which is selected as the research object in this paper, mainly consists of a hoop truss, curved front and cable net surfaces placed back-to-back across the truss with tension forces to preload the whole structure, vertical cables and a radio frequency (RF) reflective mesh stretched across the convex side of the front cable net surface. 

With the increasing interest of cable-net antenna structure in space application, a growing demand should be satisfied for its high surface accuracy. The cable-net antenna with a large size easily suffers from the effects of the particular surrounding environment, especially the thermal radiation from the sun. As the fully deployed cable-net antenna is characterized by the strong geometric nonlinearity, and the interdependency between the geometric shape and the cable tension forces, even a little disturbance from the surrounding environment may greatly change the cable tension forces and surface accuracy. Therefore, how to maintain and adjust the surface shape of cable-net antenna structures to satisfy the in-orbit performance requirements, it means the shape control, under the disturbance of the surrounding environment is extremely pivotal and challenging. 

In general, the shape control can be achieved by adjusting the lengths or tension forces of the adjustable cables. The shape control of the cable-net antenna by the direct or mode method under the small linear-elastic deformation hypothesis was carried out in [6]. The shape of the tension truss antenna was controlled by considering the sensitivity of the surface accuracy with respect to the cable length obtained by the Monte Carlo method in [7]. The multi-objective optimization methods, e.g., genetic algorithm (GA), were used in [8,9], to deal with the geometric nonlinearity problem of shape control of the cable-net antenna. Tabata et al. [10] and Du et al. [11] proposed the optimization strategies for shape control of the cable-net antenna based on the sensitivity analysis to overcome these shortcomings in [8,9]. Due to the incomplete equivalence relationship between the good RF performance and the ideal surface accuracy of the cable-net antenna, the shape control method considering the RF performance was developed in [12,13]. The direct relationship between the RF performances of a cable-net antenna with the adjustable cable lengths was investigated in [14] to be used for the shape control of cable-net antenna on the basis of the sensitivity analysis.

The smart materials and structures have been introduced into the design in recent years to improve the surface accuracy of the large cable-net antenna. The researchers at Ohio State University used the piezoelectric actuators installed on the back surface of the secondary reflector to realize the surface deformation, and thus providing different far-field pattern [15]. Wang et al. [16] and Liu et al. [17] have made a beneficial exploration in the active adjustment of the cable-net structure surface based on the smart materials. In those schemes, the piezoelectric material was utilized in the cable-net structure in the series of the truss antenna. However, the extremely high driving voltage for the piezoelectric material hampered its common aerospace engineering application in the shape control of the cable-net antenna.

Due to its shape memory effect (SME) and self-driving recoverable effect, shape memory alloy (SMA) has been paid attention gradually by engineers and scholars in active adjustment of the deployable structure in space. Hu et al. [18] presented a novel pattern reconfigurable antenna based on morphing bistable composite laminates. It was investigated experimentally the transformation between the two stable states of the proposed antenna using NiTi SMA springs. A reconfigurable axial mode helix antenna using a SMA spring actuator was studied by [19] to adjust the height of a helix antenna. A square ring antenna with a reconfigurable patch using SMA actuation was studied by [20] to maintain the impedance match while the pattern of the antenna was changed. Kalra et al. [21,22] had developed a suitable antenna actuation system, which took care of both the functionalities of beam steering and beam shaping by SMA. However, the using of SMA for the shape control of the cable-net antenna has not yet been investigated.

**Figure 1:**
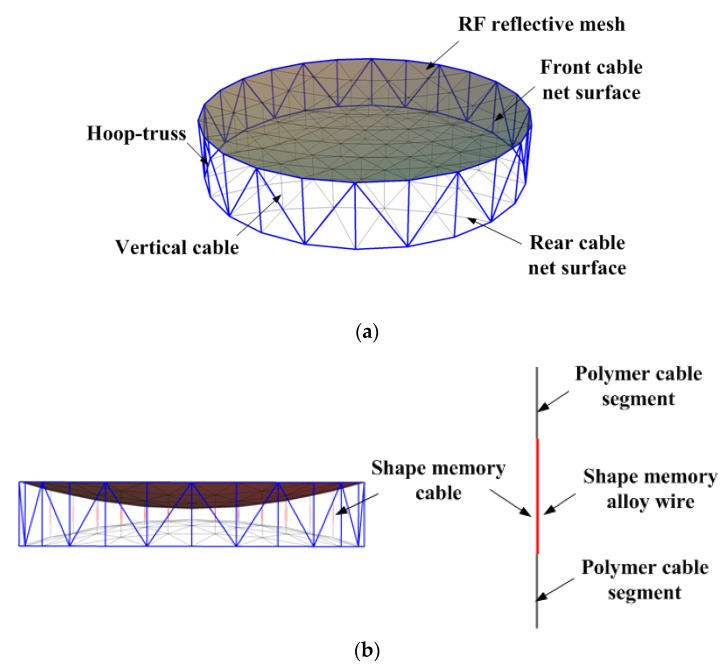
Shape memory cable-net (SMC) structure: (**a**) Composition of AstroMesh-like antenna structure and (**b**) integration mode.

In this paper, on the basis of the stiffness modeling of a 10-m diameter cable-net antenna embedded the SMA wire into each vertical cable as shown in Figure 1, named shape memory cable-net (SMC) in this paper, the shape control of the SMC is addressed using an optimization strategy for the root mean square (RMS) of the reflector surface (i.e., front cable-net surface connected with the RF reflective mesh) of SMC, which is converted into a sequential quadratic programming problem. In this scheme, an active shape control can be realized as the shape memory vertical cables are slightly shortened or lengthened under the role of temperature adjustment to achieve the required high shape accuracy for the cable-net antenna structure experienced the drastically thermal change in space.

The organization of the paper is as follows: Section 2 presents the process of the finite element modeling for SMC structure. In Section 3, a con-strained optimization model to achieve shape control, which is transformed into a sequential quadratic programming, is proposed. As a demonstration, the application of the shape control model is shown in Section 4. We finally conclude this paper with a brief summary in Section 5.

## 2. Finite Element Modeling for the SMC Structure

The force–displacement–temperature stiffness model of shape memory cables needs to be modeled on the basis of an effective SMA phase transformation constitutive model with a simple form, which ensures the calculation accuracy for the active adjustment of the surface deformation of antenna. In the last decade, some SMA constitutive models successfully predicting the occurrence of inelastic deformation during the thermo-mechanical loading can be referred in Tanaka et al. [23], Liang and Rogers [24], Brinson [25], Lagoudas et al. [26,27,28,29,30,31,32,33], Auricchio et al. [34,35,36,37] and so on. In this work, a finite element model with the one-dimensional cable element stiffness model is established to describe the elastic deformation of the polymer cable and the SME with phase transformation of the SMA wire. A one-dimensional constitutive model for the phase transformation ratcheting of superelastic SMA is cited on the basis of the Brinson work [38], and then implemented into the finite element model to analyze the active adjustment capability of the RMS surface error of the SMC. 

### 2.1. Constitutive Equation

The SMA constitutive model used here is cited from a model originally formulated by [25,38,39], which is a phenomenological constitutive model and can be expressed as:

(1) $$\sigma^{s}-\sigma_{0}^{s}=E^{s}(\xi )\varepsilon -E^{s}(\xi_{0})\varepsilon_{0}+\Theta (T-T_{0})+\Omega (\xi )\xi -\Omega (\xi_{0})\xi_{0}$$

where $\sigma^{s}$ is the axial stress of the SMA cable element, the total strain is calculated in the formulation $\varepsilon =\left(L^{s}-L_{0}^{s}\right)/L_{0}^{s}$ to approximate the real one, $E^{s}$ is the Young’s modulus, Ω is the transformation coefficient and Θ is the thermal elastic coefficient. The subscript ‘0’ indicates the initial values. Young’s modulus $E^{s}$ and the transformation coefficient Ω are the functions of the martensite volume fraction ξ, which are given as [25]:

(2a) $$E^{s}(\xi )=E_{A}+\xi (E_{M}-E_{A})$$

(2b) $$\Omega (\xi )=-\varepsilon_{L}E^{s}(\xi )$$

where $\varepsilon_{L}$ is the maximum phase transformation strain obtained from the uniaxial tension test.

In Equation (1), the total strain ε can be decomposed into an elastic strain $\varepsilon^{e}$ and an inelastic strain $\varepsilon^{in}$ with infinitesimal strain assumption:

(3) $$\varepsilon =\varepsilon^{e}+\varepsilon^{in}$$

Based on the general plasticity, the total inelastic strain $\varepsilon^{in}$ in Equation (3) is equal to the phase transformation strain $\varepsilon^{tr}$ from the stress-induced martensite phase to austenite phase and its reverse.

(4) $$\varepsilon^{in}=\varepsilon^{tr}$$

It is assumed that the transformation strain and the martensite volume fraction show a proportional relationship [25]. Then, the following relationship can be satisfied:

(5) $$\xi =\frac{\varepsilon^{tr}}{\varepsilon_{L}}$$

In the Brinson model [25,38,39], the expression forms of ξ, which is defined as the martensite volume fraction, are written as follows:

Transformation to the martensite phase:

if $T>M_{S}$ and $\sigma_{s}^{cr}+C_{M}(T-M_{s})<\sigma <\sigma_{f}^{cr}+C_{M}(T-M_{s})$:

(6a) $$\underset{A\to M}{\xi }=\frac{1-\xi_{0}^{}}{2}\cos\left\{\frac{\pi }{\sigma_{s}^{cr}-\sigma_{f}^{cr}}[\sigma -\sigma_{f}^{cr}-C_{M}(T-M_{S})]\right\}+\frac{1+\xi_{0}^{}}{2}$$

Transformation to the austenite phase:

if $T>A_{S}$ and $C_{A}(T-A_{f})<\sigma <C_{A}(T-A_{s})$:

(6b) $$\underset{M\to A}{\xi }=\frac{\xi_{0}^{}}{2}\left\{\cos[a_{A}(T-A_{s}-\frac{\sigma }{C_{A}})]+1\right\}$$

where $\sigma_{s}^{cr}$ and $\sigma_{f}^{cr}$ are the starting and finishing stresses of martensite transformation, respectively; $C_{A}$ and $C_{M}$ are the slopes for the relation between the critical phase transformation stress and temperature, respectively; $A_{s}$ and $A_{f}$ are the starting and finishing temperatures of austenite transformation, respectively; $M_{s}$ and $M_{f}$ are the starting and finishing temperatures of martensite transformation, respectively and $\xi_{0}$ is the initial martensite volume fraction, which is the martensite volume fraction prior to the current phase transformation and induced by stress load. 

The parameters $a_{A}$ and $a_{M}$ are expressed as [25]:

(7) $$a_{A}=\frac{\pi }{A_{f}-A_{s}},a_{M}=\frac{\pi }{M_{f}-M_{s}}$$

### 2.2. Finite Element Modeling for the One-Dimensional Cable Element of SMA

a) Modeling process of the SMA cable element

In this work, the SME of SMA was used to drive the produced force and deformation of SMA wire under the action of the temperature to adjust the surface accuracy of the SMC structure. In order to realize the finite element modeling of the one-dimensional cable element, the constitutive equation needs to be expressed in the incremental form. It is to solve the equivalent stiffness equation of the cable element, that is, the increment relationship between the force, temperature and deformation of the SMA wire. According to Equation (1), that relationship can be deduced as:

(8) $$F^{s}-F_{0}^{s}=E^{s}(\xi )A^{s}\frac{x}{L^{s}}-\frac{E^{s}(\xi )A^{s}}{L_{0}^{s}}x+\left[\Theta A^{s}(T-T_{0})+\Omega (\xi )\xi A^{s}-E^{s}(\xi_{0})\varepsilon_{0}A^{s}-\Omega (\xi_{0})\xi_{0}A^{s}\right]\frac{x}{L^{s}}$$

where $F^{s}=\sigma^{s}A^{s}\frac{x}{L^{s}}$ is the force vector of the SMA cable element, $F_{0}^{s}$ is the initial force vector of the SMA cable element, $A^{s}$ is the cross-sectional area of the SMA cable element, $L_{0}^{s}$ is the unstressed initial length of the SMA cable element, $L^{s}$ is the real-time length of the SMA cable element after deformation and $x/L^{s}$ is the nodal position unit vectors of the SMA cable element. 

Setting:

(9) $$Q=\Theta (T-T_{0})+\Omega \xi -E_{0}^{s}\varepsilon_{0}-\Omega_{0}\xi_{0}$$

and hence, Equation (8) can be simplified as:

(10) $$F^{s}=E^{s}(\xi )A^{s}\frac{x}{L^{s}}-\frac{E^{s}(\xi )A^{s}}{L_{0}^{s}}x+Q\frac{A^{s}x}{L^{s}}+F_{0}^{s}$$

Combining the equations,

(11a) $$\Delta \sigma =\frac{x_{}^{T}}{A^{s}L^{s}}\Delta F^{s}$$

(11b) $$\Delta \left(\frac{x}{L^{s}}\right)=\left(\frac{1}{L^{s}}I_{3\times 3}-\frac{xx_{}^{T}}{L^{s}{}^{3}}\right)\Delta x$$

(11c) $$\Delta E^{s}=\frac{dE^{s}}{d\xi }\frac{d\xi }{d\sigma }\Delta \sigma =\frac{dE^{s}}{d\xi }\frac{d\xi }{d\sigma }\frac{x_{}^{T}}{A^{s}L^{s}}\Delta F^{s}$$

(11d) $$\begin{array}{cc} \Delta Q & =\Delta \left(\Theta T+\Omega \xi \right)\\ & =\Theta \Delta T+\xi \frac{d\Omega }{dE^{s}}\frac{dE^{s}}{d\xi }\left(\frac{d\xi }{d\sigma }\Delta \sigma +\frac{d\xi }{dT}\Delta T\right)+\Omega \left(\frac{d\xi }{d\sigma }\Delta \sigma +\frac{d\xi }{dT}\Delta T\right)\\ & =\left(\xi \frac{d\Omega }{dE^{s}}\frac{dE^{s}}{d\xi }\frac{d\xi }{d\sigma }+\Omega \frac{d\xi }{d\sigma }\right)\frac{x_{}^{T}}{A^{s}L^{s}}\Delta F^{s}+\left(\xi \frac{d\Omega }{dE^{s}}\frac{dE^{s}}{d\xi }\frac{d\xi }{dT}+\Omega \frac{d\xi }{dT}+\Theta \right)\Delta T \end{array}$$

Therefore, the variational formula of Equation (10) is:

(12) $$\begin{array}{l} \Delta F^{s}=\frac{L^{s}-L_{0}^{s}}{L_{0}^{s}}\frac{dE^{s}}{d\xi }\frac{d\xi }{d\sigma }\frac{xx_{}^{T}}{L^{s}{}^{2}}\Delta F^{s}+\left(\xi \frac{d\Omega }{dE^{s}}\frac{dE^{s}}{d\xi }\frac{d\xi }{d\sigma }+\Omega \frac{d\xi }{d\sigma }\right)\frac{xx_{}^{T}}{L^{s}{}^{2}}\Delta F^{s}+\left(\frac{A^{s}}{L_{0}^{s}}x-\frac{A^{s}x}{L^{s}}\right)\frac{dE^{s}}{d\xi }\frac{d\xi }{dT}\Delta T\\ +\frac{A^{s}x}{L^{s}}\left(\xi \frac{d\Omega }{dE^{s}}\frac{dE^{s}}{d\xi }\frac{d\xi }{dT}+\Omega \frac{d\xi }{dT}+\Theta \right)\Delta T+\left(\frac{E^{s}A^{s}}{L^{s}}\frac{xx_{}^{T}}{L^{s}{}^{2}}+\frac{E^{s}A^{s}}{L^{s}}\frac{L^{s}-L_{0}^{s}}{L_{0}^{s}}I_{3\times 3}\right)\Delta x+A^{s}Q\left(\frac{1}{L^{s}}I_{3\times 3}-\frac{xx_{}^{T}}{L^{s}{}^{3}}\right)\Delta x \end{array}$$

It is rearranged further as:

(13) $$\Delta F^{s}=\frac{\beta }{\alpha }\Delta x+\frac{\gamma }{\alpha }\Delta T=K_{U}^{sma}\Delta x+K_{T}^{sma}\Delta T$$

and,

$$K_{U}^{sma}=\frac{\beta }{\alpha },\;K_{T}^{sma}=\frac{\gamma }{\alpha }$$

where $K_{U}^{sma}$ and $K_{T}^{sma}$ are the stiffness matrixes that reflect the relationship between the force and the deformation and between the force and the temperature of the SMA cable element, respectively. In the stage of phase transformation, the variables of α, β and γ are written as:

(14) $$\left\{ \begin{array}{l} \alpha =I_{3\times 3}-\frac{L^{s}-L_{0}^{s}}{L_{0}^{s}}\frac{dE^{s}}{d\xi }\frac{d\xi }{d\sigma }\frac{xx_{}^{T}}{L^{s}{}^{2}}-\left(\xi \frac{d\Omega }{dE^{s}}\frac{dE^{s}}{d\xi }\frac{d\xi }{d\sigma }+\Omega \frac{d\xi }{d\sigma }\right)\frac{xx_{}^{T}}{L^{s}{}^{2}}\\ \beta =\left(\frac{E^{s}A^{s}}{L^{s}}\frac{xx_{}^{T}}{L^{s}{}^{2}}+\frac{E^{s}A^{s}}{L^{s}}\frac{L^{s}-L_{0}^{s}}{L_{0}^{s}}I_{3\times 3}\right)+A^{s}Q\left(\frac{1}{L^{s}}I_{3\times 3}-\frac{xx_{}^{T}}{L^{s}{}^{3}}\right)\\ \gamma =\frac{A^{s}x}{L^{s}}\left(\xi \frac{d\Omega }{dE^{s}}\frac{dE^{s}}{d\xi }\frac{d\xi }{dT}+\Omega \frac{d\xi }{dT}+\Theta \right)+\left(\frac{A^{s}}{L_{0}^{s}}x-\frac{A^{s}x}{L^{s}}\right)\frac{dE^{s}}{d\xi }\frac{d\xi }{dT} \end{array}\right.$$

For the stage of the non-phase transformation, $\alpha =I_{3\times 3}$.

From Equation (2), it can be derived:

(15) $$\left\{ \begin{array}{l} \frac{dE^{s}}{d\xi }=E_{m}-E_{a}\\ \frac{d\Omega }{dE^{s}}=-\varepsilon_{L} \end{array}\right.$$

According to Equation (6), the increment formula of phase transformation driven by temperature and stress could be written as:

Transformation to the martensite phase:

(16a) $$\left\{ \begin{array}{l} \frac{\underset{A\to M}{d\xi }}{d\sigma }=-\frac{\pi \left(1-\xi_{0}^{}\right)}{2\left(\sigma_{s}^{cr}-\sigma_{f}^{cr}\right)}\sin\left\{\frac{\pi }{\sigma_{s}^{cr}-\sigma_{f}^{cr}}[\sigma -\sigma_{f}^{cr}-C_{M}(T-M_{S})]\right\}\\ \frac{\underset{A\to M}{d\xi }}{dT}=\frac{\pi C_{M}\left(1-\xi_{0}^{}\right)}{2\left(\sigma_{s}^{cr}-\sigma_{f}^{cr}\right)}\sin\left\{\frac{\pi }{\sigma_{s}^{cr}-\sigma_{f}^{cr}}[\sigma -\sigma_{f}^{cr}-C_{M}(T-M_{S})]\right\} \end{array}\right.$$

Transformation to the austenite phase:

(16b) $$\left\{ \begin{array}{l} \frac{\underset{M\to A}{d\xi }}{d\sigma }=\frac{a_{A}\xi_{0}^{}}{2C_{A}}\sin[a_{A}(T-A_{s}-\frac{\sigma }{C_{A}})]\\ \frac{\underset{M\to A}{d\xi }}{\mathrm{dT}}=-\frac{a_{A}\xi_{0}^{}}{2}\sin[a_{A}(T-A_{s}-\frac{\sigma }{C_{A}})] \end{array}\right.$$

With these equations above, the stiffness matrix $K_{U}^{sma}$ and $K_{T}^{sma}$ of the SMA cable element can be obtained. 

b) Model verification

It should be noted that the shape control function of SMC is realized by the utilization of the SME of SMA. Therefore, only the SME of SMA was verified in this section with the material parameters in [25] that means that the stress or strain responses were driven by the temperature load.

**Figure 2:**
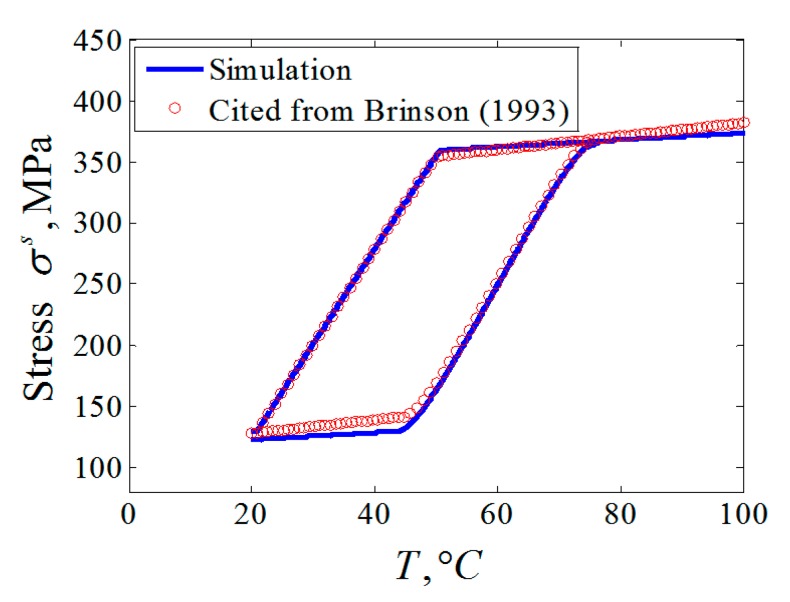
Comparison of $\sigma^{s}-T$ hysteresis between the simulation and that cited from [25].

The stiffness equation (13) and the data from Table 1 were utilized to calculate the stress response curve corresponding to the temperature variable going through *A_s_* and *A_f_* of the selected SMA. The initial states of the materials in the examples here were consistent with the original references. For example, accordingly with [25], a SMA wire with 0.5% residual martensite pre-strain was constrained to maintain that deformation as the temperature rises. It is evident that all the results presented agreed extremely well both quantitatively and qualitatively with the cited data, as shown in Figure 2.

### 2.3. Finite Element Modeling for the SMC Structure

a) Modeling process for the polymer cable element

In the shape memory cable antenna, except for shape memory materials, the design of the tensile force was implemented by the polymer cable segments. Therefore, the corresponding finite element model needed to be established. Since the polymer cable segments need to be always in the elastic stage, the one-dimensional constitutive equations are written as follows:

(17) $$\sigma {\;=\;E}_{\mathrm{cable}}\left(\varepsilon -\alpha T\right)$$

where $E_{\mathrm{cable}}$ and α depict the modulus and the thermal expansion coefficient of the polymer cable segments. 

In order to construct the finite element model of the one-dimensional cable element for polymer cable segments, the relationship between the nodal force and the element length deformation should be obtained as:

(18) $${F\;=\;A}_{\mathrm{cable}}\sigma \frac{x}{L}=\frac{E_{\mathrm{cable}}A_{\mathrm{cable}}}{L_{0}}\left(L-L_{0}\right)\frac{x}{L}-E_{\mathrm{cable}}A_{\mathrm{cable}}\alpha \left(T-T_{0}\right)\frac{x}{L}$$

where $A_{\mathrm{cable}}$ is the cross-sectional area of the polymer cable element.

Thus, the stiffness equation to express the relationship between the force, temperature and deformation is further constructed as follows:

(19) $$\left\{ \begin{array}{l} \Delta F=K_{U}^{cable}\Delta x+K_{T}^{cable}\Delta T\\ K_{U}^{cable}=\frac{E_{\mathrm{cable}}A_{\mathrm{cable}}}{L_{\mathrm{cable}}}\left[1+\alpha \left(T-T_{0}\right)\right]\frac{xx^{T}}{L_{\mathrm{cable}}^{2}}+\frac{E_{\mathrm{cable}}A_{\mathrm{cable}}}{L_{\mathrm{cable}}}\left(\frac{1}{L_{\mathrm{cable}}}-\frac{xx^{T}}{L_{\mathrm{cable}}^{2}}\right)\left[\frac{L_{\mathrm{cable}}}{L_{\mathrm{cable}0}}-1-\alpha \left(T-T_{0}\right)\right]\\ K_{T}^{cable}=-E_{\mathrm{cable}}A_{\mathrm{cable}}\alpha \frac{x}{L_{\mathrm{cable}}} \end{array}\right.$$

where $K_{U}^{cable}$ and $K_{T}^{cable}$ are the stiffness matrixes that reflect the relationship between the force and the deformation, and between the force and the temperature of the polymer cable element, respectively.

b) Finite element integration (FEI) of the SMC structure 

The equilibrium equation of the SMC can be achieved using the standard finite element assembly operation:

(20) $$\Delta F=\sum_{i_{e}}K_{ui_{e}}\Delta u_{i_{e}}+\sum_{i_{e}}K_{Ti_{e}}\Delta T_{i_{e}}=K_{U}\Delta u+K_{T}\Delta T$$

where Σ is the standard finite element assembly operator, Δ**F** is the increment of the external load vector of cable element and $\Delta u_{i_{e}}$ and $\Delta T_{i_{e}}$ are the increment of displacement vector and temperature of the node *i_e_* (*i_e_*∈*E*), respectively. Δu and ΔT are the increment of displacement vector and temperature after assembly operation respectively, and Δu=Δx is satisfied. $K_{ui_{e}}$ and $K_{Ti_{e}}$ are the stiffness matrixes of the node *i_e_*. $K_{U}$ and $K_{T}$ are the stiffness matrixes after assembly operation that are expressed as:

(21) $$\left\{ \begin{array}{l} K_{U}=K_{U}^{cable}+K_{U}^{sma}\\ K_{T}=K_{T}^{cable}+K_{T}^{sma} \end{array}\right.$$

Compared with the commonly used finite element model, the relationship among the temperature load, nodal displacement and cable length is involved in Equation (20), facilitating the analysis of active cable adjustment.

Since the nodes attached to the rim truss were assumed to be fixed, and thus these joints do not occur in displacements, Equation (21) can be rewritten in the following partitioned form after rearrangements of the nodal displacement vector Δu, the external load vector Δ**F**, and the stiffness matrices $K_{U}$ and $K_{T}$.

(22) $$\left[\begin{array}{c} \Delta F_{1}\\ \Delta F_{2} \end{array}\right]=\left[\begin{array}{cc} K_{u}^{11} & K_{u}^{12}\\ K_{u}^{21} & K_{u}^{22} \end{array}\right]\left[\begin{array}{c} \Delta u_{1}\\ \Delta u_{2} \end{array}\right]+\left[\begin{array}{c} K_{T}^{1}\\ K_{T}^{2} \end{array}\right]\Delta T$$

where $u_{1}$ and $F_{1}$ are the displacement of the internal free nodes and the external load exerted on the internal nodes, respectively; $u_{2}$ is the displacement of the boundary fixed nodes and $F_{2}$ is the reaction force by the boundary constraint, namely, the variation of the tension exerted on rim truss by cable mesh; $K_{u}^{11}$, $K_{u}^{12}$, $K_{u}^{21}$ and $K_{u}^{22}$ are the partitioned matrices of matrix $K_{U}^{}$ after rearrangement and $K_{T}^{1}$ and $K_{T}^{2}$ are the partitioned matrices of $K_{T}^{}$ after rearrangement.

Noting that $\Delta u_{2}=0$, $\Delta F_{1}=0$ (since no external load exits) during shape adjustment, substituting them into (22) leads to the relationship between nodal displacement and cable length variation as follows:

(23) $$\left\{ \begin{array}{l} \Delta u_{1}=K_{t}\Delta T\\ \Delta F_{2}=K_{u}^{21}\Delta u_{1}+K_{T}^{2}\Delta T \end{array}\right.$$

where $K_{t}=-{\left(K_{u}^{11}\right)}^{-1}K_{T}^{1}$ is the sensitivity matrix of nodal displacement increment with respect to cable temperature variation.

It can be obtained by further solution:

(24) $$\Delta F_{2}=K_{r}\Delta T$$

where,

(25) $$K_{r}=K_{u}^{21}K_{t}+K_{T}^{2}$$

## 3. Optimization Model for Shape Adjustment

### 3.1. Design Variables

Active shape adjustment of the cable net structure is achieved by altering the temperature of SMA cables. Denote the set of SMA cables by *D* and the number of its elements by *n*. For simplicity, the initial SMA cable temperature is considered as the design variable, from which the SMA cable deformation can be easily obtained and which is denoted as:

(26) $$\Delta T_{0}={\left[\Delta T_{0,j_{1}},\Delta T_{0,j_{2}},\cdots ,\Delta T_{0,j_{n}}\right]}^{T}$$

where $\Delta T_{0,j_{v}}$ (*v*=1, 2, …, *n*) is the SMA cable deformation variation of element number *j_v_* (*j_v_*∈*D*) during active shape adjustment.

### 3.2. Objective Functions

For cable net antennas, the reflector surface shaped by the cable mesh is demanded to deform to a desired shape while there is not a shape constraint on the other part of the mesh, such as the rear mesh. Here the paraboloid of the revolution of the reflector surface was considered. The divergence of reflector surface nodes with respect to a desired paraboloid needs to be minimized. Therefore, the reflector surface accuracy is the primary optimization objective in this work. 

Denoting the reflector surface nodes by the set *E*, and the number of nodes by *m*, the initial position vector of the reflector surface nodes is assumed as:

(27) $$x_{0e}={\left[x_{0,i_{1}}^{T},x_{0,i_{2}}^{T},\cdots ,x_{0,i_{e}}^{T}\right]}^{T}$$

where $x_{0,i_{e}}^{}$ (*e*=1, 2, …, *m*) is the initial position vector of node *i_e_*. The displacement vector that should be deformed during adjustment is denoted by:

(28) $$\Delta u_{e}={\left[\Delta u_{0,i_{1}}^{T},\Delta u_{0,i_{2}}^{T},\cdots ,\Delta u_{0,i_{e}}^{T}\right]}^{T}$$

where $\Delta u_{i_{e}}={\left[\Delta x_{i_{e}},\Delta y_{i_{e}},\Delta z_{i_{e}}\right]}^{T}$ is the displacement vector of node *i_e_*. Thus the nodal position after adjustment can be written as:

(29) $$x_{e}=x_{0e}+\Delta u_{e}$$

where $x_{e}={\left[x_{i_{1}}^{T},x_{i_{2}}^{T},\cdots ,x_{i_{e}}^{T}\right]}^{T}$ is the position vector of node *i_e_*. The divergence of an arbitrary node *i_e_*∈*E* with respect to a desired paraboloid is obtained as:

(30) $$\begin{array}{cc} \delta_{i_{e}}\left(x_{i_{e}}\right) & =x_{i_{e}}^{2}+y_{i_{e}}^{2}-4f\left(z_{i_{e}}+h_{0}\right)\\ & ={\left(x_{i_{e}{}_{0}}+\Delta x_{i_{e}}\right)}^{2}+{\left(y_{i_{e}{}_{0}}+\Delta y_{i_{e}}\right)}^{2}-4f\left(z_{i_{e}{}_{0}}+\Delta z_{i_{e}}+h_{0}\right) \end{array}$$

where *f* is the focal length of the paraboloid and *h*_0_ is the intercept. 

For simplicity, the surface error of reflector surface is written in the following form:

(31) $$f=\frac{1}{m}\sum_{i_{e}\in E}\delta_{i_{e}}^{2}\left(x_{i_{e}}\right)$$

Under the optimization objective above for the active adjustment of SMA cable, the reflector surface has certain self-adaptive ability to acquire high accuracy. Since the nodes at the positions where the cable mesh attaches to the rim truss can be assumed to be fixed.

### 3.3. Optimization Model

The optimization model can be summarized as: 

Find $\Delta T={\left[\Delta T_{j_{1}},\Delta T_{j_{2}},\cdots ,\Delta T_{j_{n}}\right]}^{T}$

Min $f=\frac{1}{m}\sum_{i_{e}\in E}\delta_{i_{e}}^{2}\left(x_{i_{e}}\right)$

(32) $$S.T.\;\left\{ \begin{array}{l} T_{j_{v}}\in \left(M_{f}^{j_{v}},A_{f}^{j_{v}}\right)\\ A_{s}^{j_{v}},A_{f}^{j_{v}},M_{s}^{j_{v}},M_{f}^{j_{v}}\in \left(T_{L},T_{H}\right)\\ L_{j_{v}}\leq L_{\max}\\ A_{j_{v}}^{s}\leq A_{\max}^{s},v=1,2,\cdot \cdot \cdot ,n \end{array}\right.$$

where $A_{s}^{j_{v}}$, $A_{f}^{j_{v}}$, $M_{s}^{j_{v}}$ and $M_{f}^{j_{v}}$ are the starting and finishing temperatures of austenite transformation, and the starting and finishing temperatures of martensite transformation of the $j_{v}^{\mathrm{th}}$ SMA cable element, respectively. The constraint condition on the SMA cable temperature should be restricted between $M_{f}^{j_{v}}$ and $A_{f}^{j_{v}}$. $L_{j_{v}}$ is the length of the $j_{v}^{\mathrm{th}}$ SMA cable element that should be less than the length of the vertical rim truss $L_{\max}$. The cross-sectional area of the $j_{v}^{\mathrm{th}}$ SMA cable element $A_{j_{v}}^{s}$ should be less than a given maximum area $A_{\max}^{s}$. $T_{L}$ and $T_{H}$ are the minimum and maximum temperatures determined by material properties, respectively.

## 4. Solution of the Optimization Model

### 4.1. Treatment of Objective Functions

Since the divergence between the reflector surface before the shape adjustment and a desired paraboloid is very small, the second-order terms of the displacement in Equation (30) can be omitted. Thus Equation (30) is simplified as:

(33) $$\delta_{i_{e}}\left(x_{i_{e}}\right)=2x_{i_{e}{}_{0}}\Delta x_{i_{e}}+2y_{i_{e}{}_{0}}\Delta y_{i_{e}}-4f\Delta z_{i_{e}}+x_{i_{e}{}_{0}}^{2}+y_{i_{e}{}_{0}}^{2}-4f\left(z_{i_{e}{}_{0}}+h_{0}\right)$$

Defining $A_{i_{e}}=[2x_{i_{e}{}_{0}},\;2y_{i_{e}{}_{0}},\;-4f]$, $b_{i_{e}}=x_{i_{e}{}_{0}}^{2}+y_{i_{e}{}_{0}}^{2}-4f\left(z_{i_{e}{}_{0}}+h_{0}\right)$, Equation (33) can be rewritten as:

(34) $$\delta_{i_{e}}(x_{i_{e}}^{})=A_{i_{e}}\Delta u_{i_{e}}^{}+b_{i_{e}}$$

Thus the surface error Equation (31) is rewritten as:

(35) $$\begin{array}{l} f=\frac{1}{m}\sum_{i_{e}\in E}\delta_{i_{e}}^{2}\left(x_{i_{e}}\right)=\frac{1}{m}\sum_{i_{e}\in E}{\left[A_{i_{e}}\Delta u_{i_{e}}^{}+b_{i_{e}}\right]}^{2}\\ \begin{array}{cc} & =\Delta u_{e}^{T}A^{T}A\Delta u_{e}^{}+2B^{T}A\Delta u_{e}^{}+B^{T}B \end{array} \end{array}$$

where $A=\frac{1}{\sqrt{m}}\mathrm{diag}\left(\;[A_{1},\;A_{2},\;\cdots ,\;A_{m}]\;\right)$, and $B=\frac{1}{\sqrt{m}}{\left[b_{1},\;b_{2},\;\cdots ,b_{m}\right]}^{T}$.

### 4.2. Iterative Procedure

**Figure 3:**
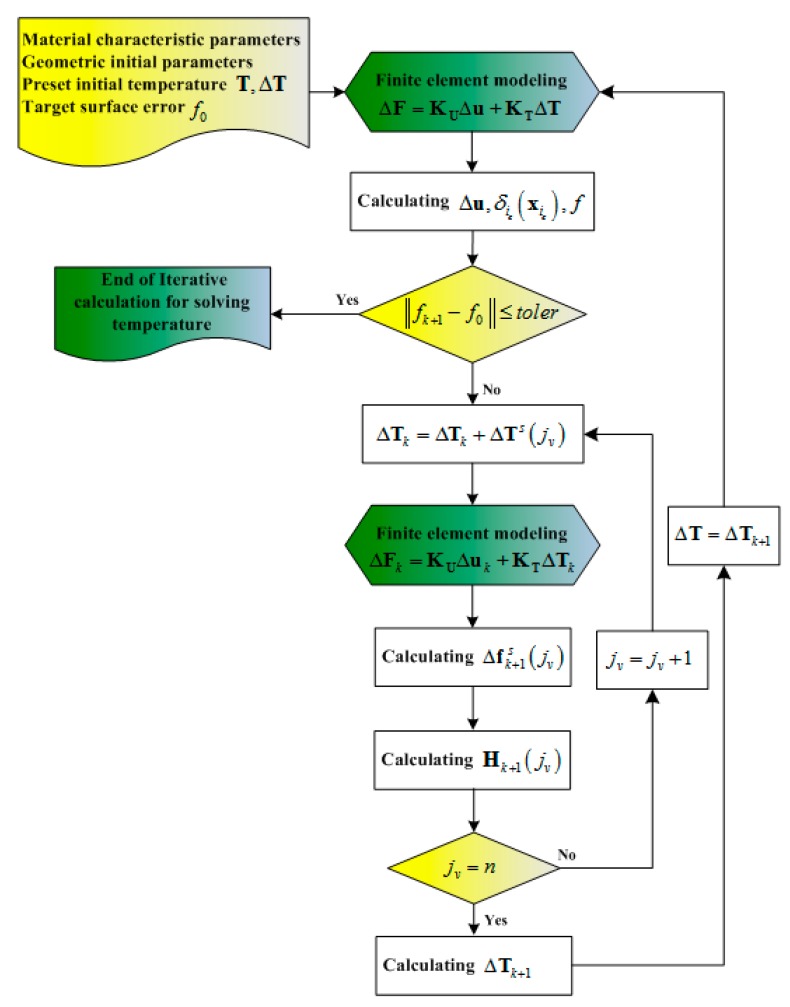
Flow chart of the iterative procedure to determine the applied temperature on shape memory alloy (SMA) to obtain the target surface error.

The shape adjustment procedure for SMC should be converted into a sequential quadratic programming problem that is an iterative procedure due to the geometric nonlinearity of SMC. Figure 3 shows the flow chart of the iterative procedure to determine the applied temperature on SMA to obtain the target surface error, which can be implemented as follows:

1) Given the initial state of the (*k*+1)*^th^* iterative substep: $\Delta T^{s}$, $T_{k}$ and $f_{k}^{}$, where $\Delta T^{s}$ is the designated temperature increment vector of the SMA cables for the optimization calculation, $T_{k}$ is the temperature vector of the SMA cables of *k^th^* iterative substep, and $f_{k}^{}$ is the surface error of *k^th^* iterative substep.
2) Therefore, the temperature vector of the SMA cables of the (*k*+1)*^th^* iterative substep for the optimization calculation can be given as: $T_{k+1}^{s}=T_{k}+\Delta T^{s}$.
3) The surface error vector corresponding to the $j_{v}^{\mathrm{th}}$ SMA cable element is given as: $\Delta f_{k+1}^{s}\left(j_{v}\right)=f_{0}-f_{k+1}^{s}\left(j_{v}\right)$ under the temperature increment $\Delta T^{s}\left(j_{v}\right)$ of the $j_{v}^{\mathrm{th}}$ SMA cable element, where $f_{0}$ is the objective surface error.
4) These results were used to create the small perturbation influence matrix for optimization as: $H_{k+1}^{}\left(j_{v}\right)=\frac{\Delta f_{k+1}^{s}\left(j_{v}\right)}{\Delta T^{s}\left(j_{v}\right)}=\frac{f_{0}-f_{k+1}^{s}\left(j_{v}\right)}{\Delta T^{s}\left(j_{v}\right)}$.
5) The least squares solution for the temperature vector of the SMA cables of the (*k*+1)*^th^* iterative substep can be calculated by $\Delta T_{k+1}=-{\left[H_{k+1}^{T}H_{k+1}^{}\right]}^{-1}H_{k+1}^{T}f_{k}^{}$.
6) Finally, the temperature vector of the SMA cables of the (*k*+1)*^th^* iterative substep for the surface error $f_{k+1}^{}$ is given as: $T_{k+1}^{}=T_{k}+\Delta T_{k+1}^{}$. The iterative termination conditions were set as: $\left\|f_{k+1}^{}-f_{0}^{}\right\|\leq toler$.

## 5. Numerical Simulation

**Figure 4:**
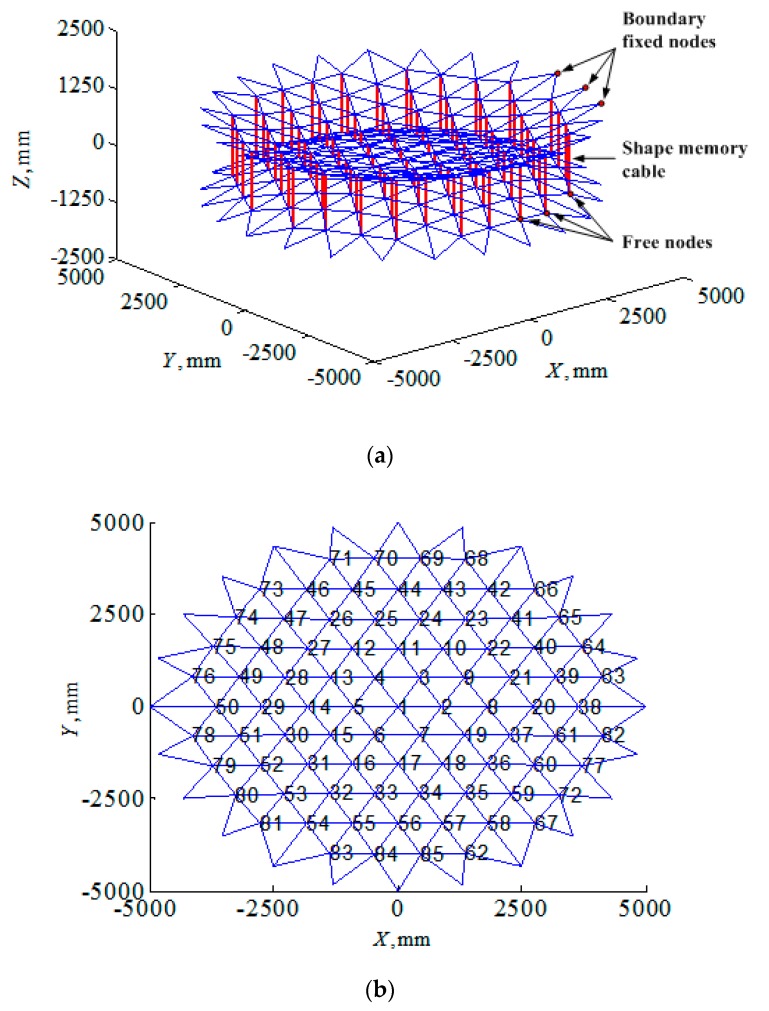
SMC in the deployable state in this work: (**a**) Isometric view and (**b**) free nodes numbering of the front reflector surface.

In this section, the proposed shape control procedure would be applied to a 10-m diameter SMC (shown in Figure 4a) to illustrate its feasibility. The shape accuracy of the parabolic reflector surface controlled by SMA cables was estimated by RMS error. The antenna specifications are described as follows:

Diameter of aperture: 5000 mm;

Focal length of front cable-net surface: 8000 mm;

Number of free nodes: 170;

Number of fixed nodes: 48;

Number of cables: 552;

Number of shape memory cables: 85;

Type of facet: Triangular;

Elastic modulus of cable: 20 GPa;

Diameter of cross-section of polymer cable: 1 mm.

The material parameters of the SMA actuators as shown in Table 1 was utilized for the numerical simulation in this work. The numbering scheme of the free nodes of the front reflector surface is shown in Figure 4b. The element numbering could be assigned randomly.

**Figure 5:**
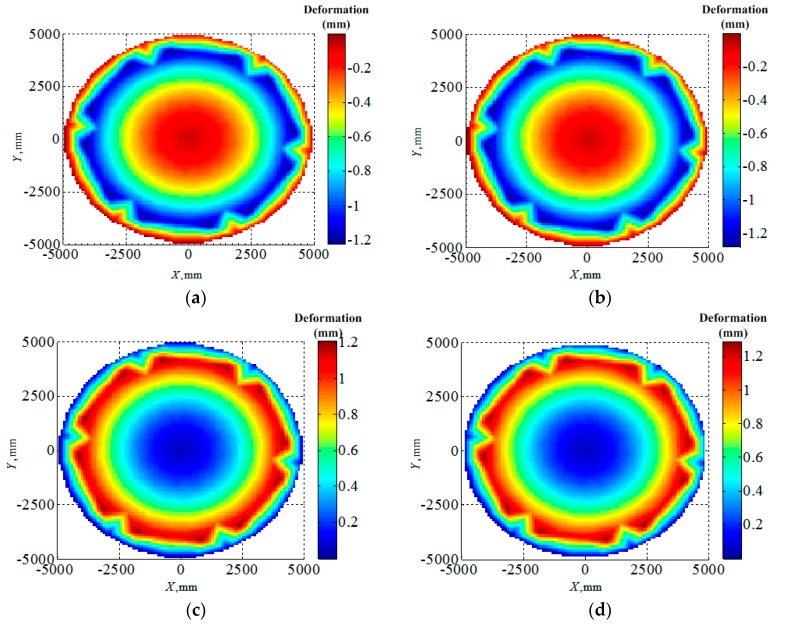
Comparison of the thermal deformation computed by the FEI method in this work and ANSYS: (**a**) Thermal deformation by FEI under 140 °C, (**b**) thermal deformation by ANSYS under 140 °C, (**c**) thermal deformation by FEI under −100 °C and (**d**) thermal deformation by ANSYS under −100 °C.

To confirm the validity of the finite element modeling of the cable-net structure, the commercial software ANSYS (Ansys 15.0) was employed with the reference temperature 20 °C. Figure 5 shows the node displacements of the cable-net antenna reflector surface with whole polymer cables, which meant no SMA materials for the calculated cable-net structure, for two different terminal temperatures of 140 °C and −100 °C. It is evident that the node displacements obtained by the FEI method in this work agreed very well with those computed by ANSYS, and the maximum difference by these two approaches was within 6%. 

Based on the effective stiffness model of the SMA wire and the FEI method, the simulations based on the finite element model of SMC with 10-m diameter were carried out in the following work. It should be noted that the current work was to investigate the active adjustable capability of the SMC for the optimal design of RMS. Therefore, it was assumed that the environmental temperature load was only applied on the polymer cables of SMC (i.e., cables belonged to the front and rear cable-net surfaces). On the other hand, the temperatures of the SMA cables of SMC were controlled under the direction of the optimal design model.

After the environment temperature loading for the SMC model, it could be found that the RMS changed from near-zero to 1.1 mm and 2.24 mm, and the focal length changed from 8000 mm to 7996.6 mm and 8006.1 mm, for the high environment temperature and the low environment temperature, respectively. According to the focal length change, it could be found that the vertical SMA cables were shortened under the high environment temperature. Therefore, the SMA cable was expected to lengthen along with the decreasing of the tension stress during the optimal design for this environment temperature. On the contrary, the SMA cable should shorten along with the increasing of the tension stress for the low environment temperature.

From the $\sigma^{s}-T$ hysteresis curve in Figure 2, the decreasing of the tension stress occurred along with the martensite forward phase transformation under the reduction of the control temperature of the SMA wire, with the high initial control temperature and the low initial martensite volume fraction. On the contrary, the increase of the tension stress along with the martensite reverse phase transformation could be realized under the increase of the control temperature of the SMA wire with the low initial control temperature and the high initial martensite volume fraction. According to Equations (1) and (6a), the initial stress and the initial martensite volume fraction of the single SMA wire can be calculated by the following equations:

(36a) $$\sigma_{0}^{s}=\frac{F_{0}^{s}}{A^{s}}$$

(36b) $$\xi_{0}^{}=\frac{1}{2}\cos\left\{\frac{\pi }{\sigma_{s}^{cr}-\sigma_{f}^{cr}}[\sigma_{0}^{s}-\sigma_{f}^{cr}-C_{M}(T_{0}-M_{S})]\right\}+\frac{1}{2}$$

(36c) $$\varepsilon_{0}=\frac{\sigma_{0}^{s}}{E^{s}(\xi_{0})}+\varepsilon_{L}\xi_{0}$$

where $F_{0}^{s}$ is the initial tension force of the SMA cable of SMC that is calculated by the force density method [40]. Based on the analyses above, the initial control temperatures were set as 38 °C and 75 °C under the high and low environment temperatures. The initial tension stress of the SMA wire was identical for both cases, and within the range of 125–156 MPa for all SMA wires calculated by Equation (36a). The initial martensite volume fraction were calculated by Equation (36b) as obtained within the range of 0.003–0.26 for the high environment temperature, and within the range of 0.035–0.61 for the low environment temperature. Based on the calculation results, the initial strain of the SMA wire could be obtained by Equation (36c), and within the range of 0.002–0.02 and 0.004–0.043 for the high and low environment temperatures. 

**Figure 6:**
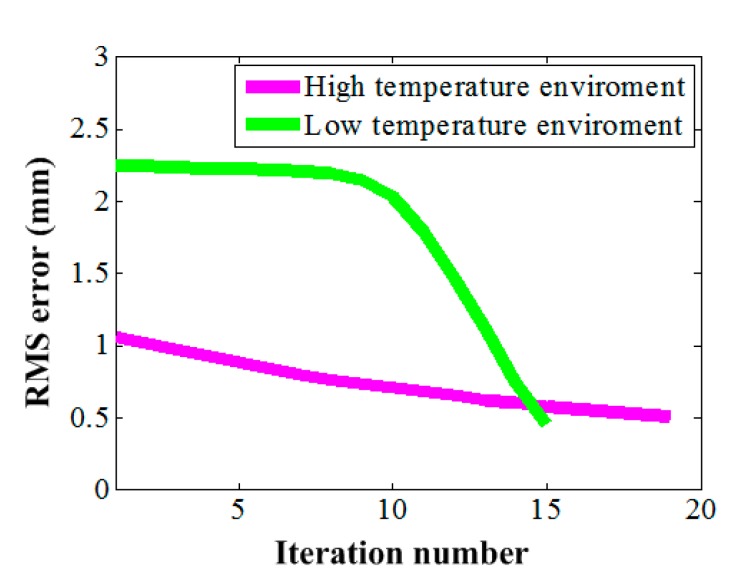
Surface root mean square (RMS) error during adjustment.

The shape adjustment procedure converged after 19 and 15 iterations for the high and low environment temperatures, respectively. The RMS errors were reduced from the initial values 1.1 mm and 2.24 mm to 0.49 mm and 0.46 mm as shown in Figure 6 for the high and low environment temperatures, respectively. These were very high surface accuracies for the most space antennas employed currently operating at the L to Ka bands. For example, the preferable surface accuracy working at 15 GHz (Ku band) is 0.4 mm (RMS) [11]. 

**Figure 7:**
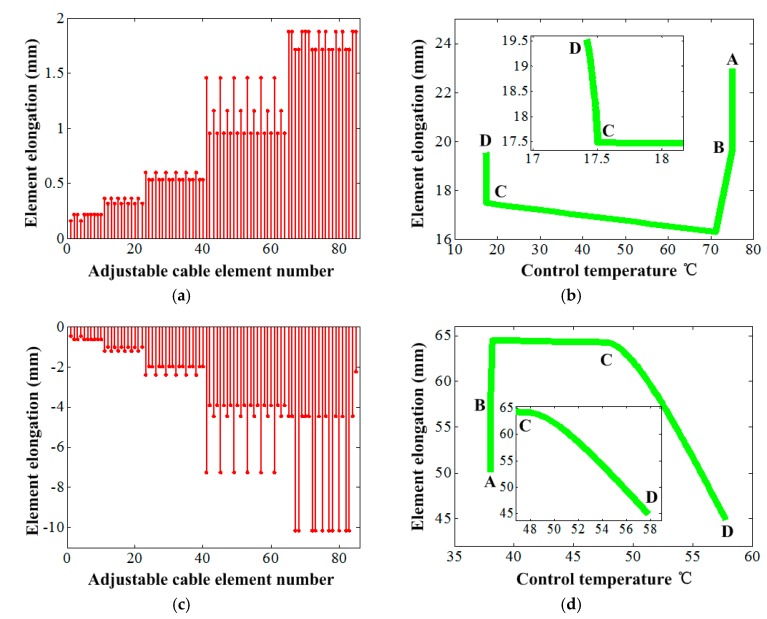
Element elongation under the control temperature: (**a**) Variations of all elements of vertical cables under environment temperature 140 °C, (**b**) single element change under environment temperature 140 °C, (**c**) variations of all elements of vertical cables under environment temperature −100 °C and (**d**) single element change under environment temperature −100 °C.

The whole elongation variations of adjustable SMA cable elements are shown in Figure 7a under the high environment temperature. It can be found that all SMA cable elements lengthened from the minimum value 0.16 mm to the maximum value 1.88 mm, the adjustment magnitude gradually increased from the elements of the central region to the outside ones. The elongation change process of an element (e.g., SMA cable element with node number 80) with the control temperature is shown in Figure 7b. In this figure, the points A, B, C and D present the initial state of the selected element, the state of the selected element placed in environment temperature, the phase transformation start point and the calculation convergent point of the selected element. It could be found that the length of the selected element became short due to the high environment temperature. The optimization iteration calculation experienced from point B, through C, to D by the optimization model as described in Section 3.3, which included the non-phase transformation process (i.e., from point B to C) and the phase transformation process (i.e., from point C to D). Figure 7c depicts the whole elongation variations of adjustable SMA cable elements under the low environment temperature. All SMA cable elements were adjusted to be shortened from the minimum value 0.47 mm to the maximum value 10.15 mm. The adjustment magnitudes of those elements gradually also increased from the elements of the central region to the outside ones, whose rule was similar to that of the high environment temperature. Figure 7d depicts the elongation change process of the selected SMA cable element with node number 80 under the control temperature in the case of the low environment temperature. In Figure 7d, the control temperature gradually increased with the numbering of the iteration step that was contrary to that shown in Figure 7b. At the same time, the relationship between the element elongation and the control temperature at the phase transformation stage (i.e., from point C to D) in Figure 7d shows obviously a slower relative change of each other than that under the low environment temperature as shown in Figure 7b.

**Figure 8:**
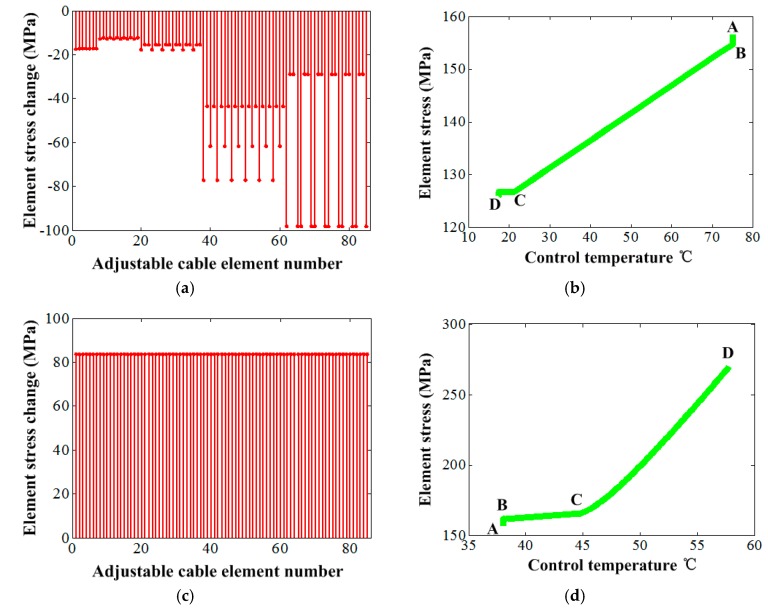
The element stress change under the control temperature: (**a**) Variations of all elements of vertical cables under environment temperature 140 °C, (**b**) single element change under environment temperature 140 °C, (**c**) variations of all elements of vertical cables under environment temperature −100 °C and (**d**) single element change under environment temperature −100 °C.

Figure 8 gives the element stress change under the control temperature. For the high environment temperature, the rule of the adjustment magnitudes of the element stress of those adjustable SMA cable elements was similar to that of the element elongation change case, which showed a gradual increase from the elements of the central region to the outside ones from 12.1 MPa to 98.1 MPa, as shown in Figure 8a. However, for the low environment temperature, the adjustment magnitudes of the stress of those elements were identical completely as 83.7 MPa, as shown in Figure 8c. The stress adjustment of the selected element with node 80 presented a clear change at the materials non-phase transformation stage, and showed near no change at the phase transformation stage for the high environment temperature, as shown in Figure 8b. On the contrary, the change mainly occurred at the materials phase transformation stage, and kept approximate stable at the non-phase transformation stage for the low environment temperature, as shown in Figure 8d.

**Figure 9:**
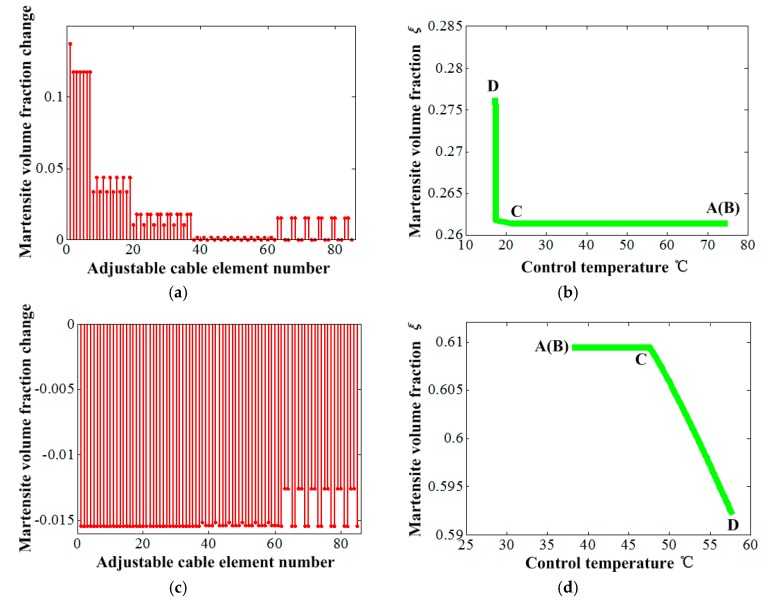
The martensite volume fraction change under the control temperature: (**a**) Variations of all elements of vertical cables under environment temperature 140 °C, (**b**) single element change under environment temperature 140 °C, (**c**) variations of all elements of vertical cables under environment temperature −100 °C and (**d**) single element change under environment temperature −100 °C.

Figure 9 shows the element martensite volume fraction change under the control temperature. It can be found that the adjustment magnitudes of the element martensite volume fraction of the SMA cable elements for the high environment temperature show a gradual decrease from the elements of the central region to the outside ones from 0 to 0.14 shown in Figure 9a, which presents an opposite rule with that shown in Figure 6 and Figure 7. However, for the low environment temperature, the adjustment magnitudes of the martensite volume fraction of those elements show nearly identical from 0.013 to 0.015, as shown in Figure 9c. The martensite volume fraction adjustment of the selected element with node 80 shows dramatic growth within a very little temperature increment at the phase transformation stage for the high environment temperature, as shown in Figure 9b. On the contrary, it changed much more gently at the materials phase transformation stage for the low environment temperature, as shown in Figure 9d.

## 6. Conclusions

Shape adjustment of the cable-net structure activating by the SMA incorporated into the vertical cable was investigated in this paper. The constructed equivalent stiffness equation of the SMA could effectively express the relationship between the force, temperature and deformation of the materials. The optimization method was verified to be capable of improving the in-orbit surface accuracy of the cable-net antenna under the influence of the space environment temperature. The setting of the initial parameters of the materials was confirmed to be very important to achieve the optimization of the in-orbit surface accuracy of the cable-net structure. The shape adjustment results show that the region with the larger thermal deformation obtained the larger adjustment magnitude of the SMA. A little bit of change of the martensite volume fraction was enough to obtain the required deformation of the materials. However, the active adjustment strategy of surface accuracy of the SMC needs a large amount of space energy that is a practical problem to be realized. Therefore, the passive adjustment strategy of surface accuracy of the SMC just relying on the space thermal radiation may be a valuable objective in future work.

## Figures and Tables

**Table 1:** Material parameters for the model verification [25].

$E_{A}^{}=67\;\mathrm{GPa}$	$E_{M}^{}=26.3\;\mathrm{GPa}$	$\nu_{A}^{}=0.3$	$\nu_{M}^{}=0.3$	$\varepsilon_{L}^{}=0.067$
$M_{f}=9\;{}^{\circ}C$	$M_{s}=18.4\;{}^{\circ}C$	$A_{s}=34.5\;{}^{\circ}C$	$A_{f}=49\;{}^{\circ}C$	Θ=0.55 MPa/°C
$C_{M}=8\;\mathrm{MPa}/{}^{\circ}C$	$C_{A}=13.8\;\mathrm{MPa}/{}^{\circ}C$	$\sigma_{s}^{cr}=100\;\mathrm{MPa}$	$\sigma_{f}^{cr}=170\;\mathrm{MPa}$	-
